# Improved assessment of multiple sclerosis lesion segmentation agreement via detection and outline error estimates

**DOI:** 10.1186/1471-2342-12-17

**Published:** 2012-07-19

**Authors:** David S Wack, Michael G Dwyer, Niels Bergsland, Carol Di Perri, Laura Ranza, Sara Hussein, Deepa Ramasamy, Guy Poloni, Robert Zivadinov

**Affiliations:** 1 Buffalo Neuroimaging Analysis Center, Dept. of Neurology, University at Buffalo, State University of New York at Buffalo, Buffalo, NY, USA; 2 Department of Nuclear Medicine, University at Buffalo, State University of New York at Buffalo, Buffalo, NY, USA; 3 Department of Neuroradiology, IRCCS, C. Mondino, University of Pavia, Pavia, Italy; 4 The Jacobs Neurological Institute, Dept. of Neurology, University at Buffalo, State University of New York at Buffalo, Buffalo, NY, USA; 5 Buffalo Neuroimaging Analysis Center, Jacobs Neurological Institute, State University of NY at Buffalo, 100 High St., Buffalo, NY, 14203, USA

**Keywords:** Multiple sclerosis, Detection and outline error estimates, Rater agreement, Operator agreement, Metric, Jaccard Index, Similarity index, Measure, Index, Kappa, Lesion, MRI, ROI

## Abstract

**Background:**

Presented is the method “Detection and Outline Error Estimates” (DOEE) for assessing rater agreement in the delineation of multiple sclerosis (MS) lesions. The DOEE method divides operator or rater assessment into two parts: 1) Detection Error (DE) -- rater agreement in detecting the same regions to mark, and 2) Outline Error (OE) -- agreement of the raters in outlining of the same lesion.

**Methods:**

DE, OE and Similarity Index (SI) values were calculated for two raters tested on a set of 17 fluid-attenuated inversion-recovery (FLAIR) images of patients with MS. DE, OE, and SI values were tested for dependence with mean total area (MTA) of the raters' Region of Interests (ROIs).

**Results:**

When correlated with MTA, neither DE (ρ = .056, p=.83) nor the ratio of OE to MTA (ρ = .23, p=.37), referred to as Outline Error Rate (OER), exhibited significant correlation. In contrast, SI is found to be strongly correlated with MTA (ρ = .75, p < .001). Furthermore, DE and OER values can be used to model the variation in SI with MTA.

**Conclusions:**

The DE and OER indices are proposed as a better method than SI for comparing rater agreement of ROIs, which also provide specific information for raters to improve their agreement.

## Background

Multiple operators are often used to draw regions of interest (ROIs) on medical images when the workload would be too great for a single operator. When using multiple operators, it is desirable to have the ROIs from each to be similar. There are multiple measures available to assess inter-rater variability, such as Kappa, Jaccard’s Index (JI), Similarity Index (SI), Hausdorff Distances, Conformity and Sensibility, etc. [1-6]. We want to be able to assess an operator’s (or automated method’s) ability to create lesion ROIs, using the ROIs they created. However, for any assessment we should consider whether some test scans are easier or harder than others to achieve good measured agreement on. An ideal measure would solely reflect the operator’s ability, and not the difficulty of the underlying test scans.

One of the original and common results of multiple sclerosis lesion segmentation is the determination of the total lesion volume for an individual subject. A center may validate operators by their ability to draw ROIs that are in agreement with the overall lesion volume of a gold standard analysis. Fortunately, this intra-observer agreement was not found to be significantly correlated with lesion volume [7]. However, this only assesses an operator’s ability to calculate total lesion volume; it does not make a strong statement about the ability of the operator to produce ROIs which agree with a set of ground truth ROIs, since whether small lesions are even marked has little impact. For this purpose, Valmet [8] or STAPLE (Simultaneous Truth and Performance Level Estimation) [9] would be a better choice.

SI is perhaps the most commonly used index and is defined as two times the area of the intersection of the raters' ROIs, divided by the sum of the area of the raters ROIs. Unfortunately, the lesion burden (total lesion volume) of MS patients whose scans are used for the comparison of operators' ROIs has been observed to affect an operator's or algorithm's agreement level, with an index such as SI [10-14]. In short, scans depicting high lesion load are “easier” than scans depicting low lesion load for operators to achieve high SI agreement. This is partly because scans depicting high lesion burden will typically have unambiguous large lesions that are hard for raters to disagree in marking. Hence, it is difficult to assess raters or automated methods against one another with SI if they are evaluated on different sets of scans having different lesion burdens. This is the usual case when results are published in the literature, using that center’s data set. Udupa et al. [3,4] used measures which included an agreement by the raters to mark the same region in their ROIs (*detection*), and an agreement with respect to how those regions were *outlined*. Our approach will re-express SI in terms of detection and outline error measurements so that SI's dependency on lesion burden can be well understood.

Our belief is that, unlike SI, our detection and outline error measures won't show a strong dependency on the underlying lesion burden of the scans used in comparing a pair of raters. While scanner issues such as the type of scan acquired and scan quality will still have an impact on operator agreement, reducing the influence of lesion burden (a study population condition) will represent a major improvement for the evaluation of operators. Our development and testing will be performed with operators measuring hyperintense fluid-attenuated inversion-recovery (FLAIR) MRI lesions associated with MS. However, with appropriate testing our method should be directly applicable to ROIs used to measure other hyper- or hypo-intensities, and can also be used to compare a single rater or algorithm to a gold standard. Additionally, we present two graphs for use in comparing a pair of raters. The first compares the raters detection errors (differences) based on region size. The other compares the relative differences between raters in the outline of a lesion.

### Theory

*Similarity Index:* SI is commonly defined as 2 times the area of the intersection of the raters' ROIs, divided by the sum of the area of the raters ROIs:

(1) $$SI(R1,R2)=\frac{2|R1\cap R2|}{\left|R1|+|R2\right|}$$

where | R1 ∩ R2| represents the area of the intersection of rater 1 and rater 2's ROIs, |R1| represents the area of rater 1's ROIs, and |R2| represents the area of rater 2's ROIs. Our approach is 2-dimensional to be reflective of how a human operator views and marks the images. Letting MTA (Mean Total Area) of the two raters' ROIs equal 1/2(|R1| + |R2|), we express SI as:

(2) $$SI(R1,R2)=\frac{\left|R1\cap R2\right|}{MTA}$$

**Figure 1:**
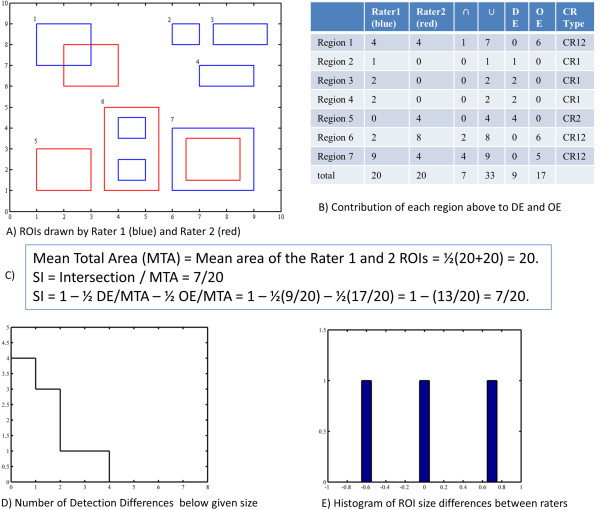
**(A): illustrates ROIs drawn from two different raters.** Regions 1, 6, and 7 are examples where both raters agree in the detection of a lesion but disagree on how it should be outlined; we designate these regions as type CR12. Regions 2, 3, and 4 are examples where only rater 1 (blue) drew an ROI, thus we designate these regions as type CR1. Finally, only rater 2 (red) drew an ROI for region 5, thus we designate this region as type CR2. (**B**): (table). For each region depicted in Figure 1 (**A**), we calculate sizes of rater 1’s ROIs, rater 2’s ROIs, the intersection of their ROIs, and the union of their ROIs. For regions where only one of the raters drew an ROI, (types CR1 and CR2) we calculate the contribution to DE and not OE. In this case, DE will equal the size of the only drawn ROI. For regions where both raters drew an ROI (type CR12), we calculate the contribution to OE and not DE. In this case, OE will equal the area of the difference between the union and intersection of the ROIs. The bottom row shows the total area for the ROIs drawn by both raters, the total area of intersection and the union of the raters' ROIs, together with the DE and OE errors for the set of ROIs. These values can be used to calculate SI directly, or in terms of DE and OE. (**C**): Calculation of SI from traditional equation and in terms of DE and OE. (**D**): A plot of the total number of regions of types CR1 and CR2 smaller than a given area is shown for the ROIs displayed in Figure 1 (**A**). This plot can be useful in determining the average number of detection differences above a specified lesion size threshold. For instance, we have 1 detection error for ROIs larger than 3. We would expect two errors between raters if we threshold their ROIs to be larger than 2. (**E**): is the histogram of (Area of Rater 1’s ROI – Area of Rater 2’s ROI) / Area of the Union of Rater 1 and Rater 2 ROIs (i.e., (column 3 – column 2) / column 5, from the table (B), for regions of type C12. If more regions were used, this graph would begin to form a bell-shaped curve. Regions where experts closely agree in outlining a curve would contribute to the center of the graph, while a large disagreement would contribute to one of the edges.f

If the ROIs from both raters are overlaid on the same image slice, the union of the ROIs will typically mark several connected regions on the image slice. We classify each connected region from the union of the raters' ROIs as one of three types: CR1, CR2, or CR12, depending on whether a region is from an ROI(s) only from rater 1, only from rater 2, or a combination of from both raters, respectively. In Figure 1 (A), we present several sample ROIs and provide classifications of connected regions in Figure 1 (B).

*Detection Error and Outline Error:* Our method for measuring rater agreement calculates the sum of the pixels that were marked by only one rater. We define Detection Error (DE) as the total area of all CR1 and CR2 regions:

(3) $$DE=\sum_{cr\in CR1orCR2}|cr|\text{,}$$

where |*cr*| represent the area of the connected region, *cr*; and *cr* ∈ CR1 or CR2 represents the set of connected regions that can be labeled as either CR1 or CR2. We define Outline Error (OE) as the total difference between the union and intersection of the CR12 ROIs:

(4) $$OE=\sum_{cr\in C12}|cr|-|R1(cr)\cap R2(cr)|\text{,}$$

where |R1(*cr*)| and |R2(*cr*)| represent the areas of rater 1 and rater 2's ROIs within *cr*, respectively. For a connected region, either DE or OE is calculated but not both. In Figure 1 (B) we also calculate DE and OE for the ROIs in Figure 1 (A). Simple algebra relates DE and OE with SI:

(5) $$SI(R1,R2)=1-\left(\frac{1}{2}\right)\frac{OE}{MTA}-\left(\frac{1}{2}\right)\frac{DE}{MTA}$$

In Figure 1 (C) we demonstrate the calculation of SI using the original equation and our equation using DE and OE.

While it is not clear whether DE will vary with MTA, we expect that OE will increase with the number or average size of ROIs for a scan (i.e., an increase in MTA). Therefore, we define the Outline Error Rate (OER) as *OER = OE/MTA*, and express SI as:

(6) $$SI(R1,R2)=1-\left(\frac{1}{2}\right)OER-\left(\frac{1}{2}\right)\frac{DE}{MTA}$$

Our assumption is that DE and OER are descriptive measures of the raters' agreement level across images for a full range of lesion burdens, and can be considered constants. Hence, this assumption implies that SI is not constant across a range of lesion burdens. Letting *mean*DE and *mean*OER represent the mean value of DE and OER, respectively, we can estimate SI for images with varying lesion burdens (measured in terms of MTA):

(7) $$SI_{\mathrm{estimate}}\left(R1,R2\right)=1-\left(\frac{1}{2}\right)meanOER-\left(\frac{1}{2}\right)\frac{meanDE}{MTA}$$

The goal of calculating *mean*OER and *mean*DE is to provide measures for rater assessment that are better than current indices by virtue of having reduced dependence on MTA.

SI is closely related to Kappa and JI. SI can be shown to be the limit of Kappa [4] when the agreement between operators includes an increasingly large number of voxels not marked as a lesion by either rater. Furthermore, SI can be expressed in terms of JI as SI = 2 JI / (1 + JI), when JI is defined as equaling the size of the intersection divided by the size of the union of the raters' ROIs. The relationship between SI, JI, and Kappa is nearly linear for the general range we expect in comparing operators’ ROIs, which typically have SI values between .35 and .85.

*Cumulative Detection Error:* We expect operators to have a greater number of detection disagreements determining whether small hyperintense regions should be marked as lesions, rather than larger regions. We create a Cumulative Detection Error graph to answer the question: "How many ROIs were drawn by only one rater with the ROI sizes greater than a given threshold?" The graph is the number of CR1 and CR2 regions with ROI areas greater than a given area threshold, and decreases with increasing threshold values. In Figure 1 (D), we plot the total number of detection errors for the ROIs in Figure 1 (A). Similarly, we could plot the Cumulative Detection Error graphs for CR1 and CR2 separately. Together, these graphs would indicate if one rater was more lenient or strict than the other rater in determining whether a small hyperintense region should be marked as a lesion.

*Outline Error Distribution:* We are also concerned with whether one rater consistently creates smaller or larger ROIs than the other. Our approach is to plot the histogram of $\frac{\left(\left|R2|-|R1\right|\right)}{\left|R1\cup R2\right|}$ for all CR12 regions, which we term the Outline Error Distribution graph. Figure 1 (E) shows the Outline Error Distribution graph for the ROIs in Figure 1 (A), which had only 3 CR12 regions. A more typical usage would have hundreds or thousands of CR12s. An ideal distribution would be a thin peak located at 0.

## Methods

### Subjects and MR acquisition

FLAIR scans from 17 participants, aged 18–80 years, with Expanded Disability Status Scores (EDSS) [15] (0–8.5) fulfilling the criteria for clinically definite MS [16] were analyzed by two raters. Informed consent was obtained from all participants, and the study was approved by the University at Buffalo's Health Science Institutional Review Board. Scans were performed on a 3 T GE Signa Excite HD 12.0 Twin Speed 8-channel scanner (General Electric, Milwaukee, WI) using a GE multi-channel head and neck coil. FLAIR scans had TE = 120 ms and TR = 8500 ms. Image voxel size was .94×.94×3 mm^3^. A full description of the scanning protocol was described in a recently published study [17]. Both raters were physicians with several years of MS research experience, but had only three months experience at our lab at the time of the experiment. Lesion contouring was performed with JIM 4.0 software (Xinapse Systems Limited, Aldwincle, U.K.), according to established lab guidelines. Hyperintense lesions were outlined using JIM's semi-automated contouring tool, which allows an operator to fully specify a lesion outline by clicking the mouse only near the edge of the lesion; a few outlines (< 2%) required manual editing to achieve a proper lesion contour. Software was written in-house using MATLAB (Natick, MA).

### Processing

For each image slice analyzed by both raters, the closed-path line segment JIM ROIs are used to form image masks. The lesion outlines are converted to an image mask by up-sampling the image by a factor of 5 in both the x and y directions to minimize the possibility that a single closed path ROI forms multiple regions, which can occur if an ROI has a narrow section (less than one pixel) between two larger sections. A binary “or” of the two masks is used to form the union. Each distinct separate region of the union “or” operation is referred to as a “Connected Region” (CR). The connected regions are labeled as CR1, CR2, or CR12, based on whether the CR was form by the ROIs of rater 1, rater 2, or raters 1 and 2.

### Evaluation criteria

DE, OE, OER, and SI were compared to MTA both graphically and by Spearman rank correlation. The mean values of OER and DE were used to express SI as a function of MTA, and compared to the true values of SI both graphically and using Pearson linear correlation. We also compare our fit of SI to linear and quadratic fits of SI as a function of MTA. Finally, we demonstrate the utility of the Outline Distribution Error and Cumulative Detection Error graphs.

## Results

**Figure 2:**
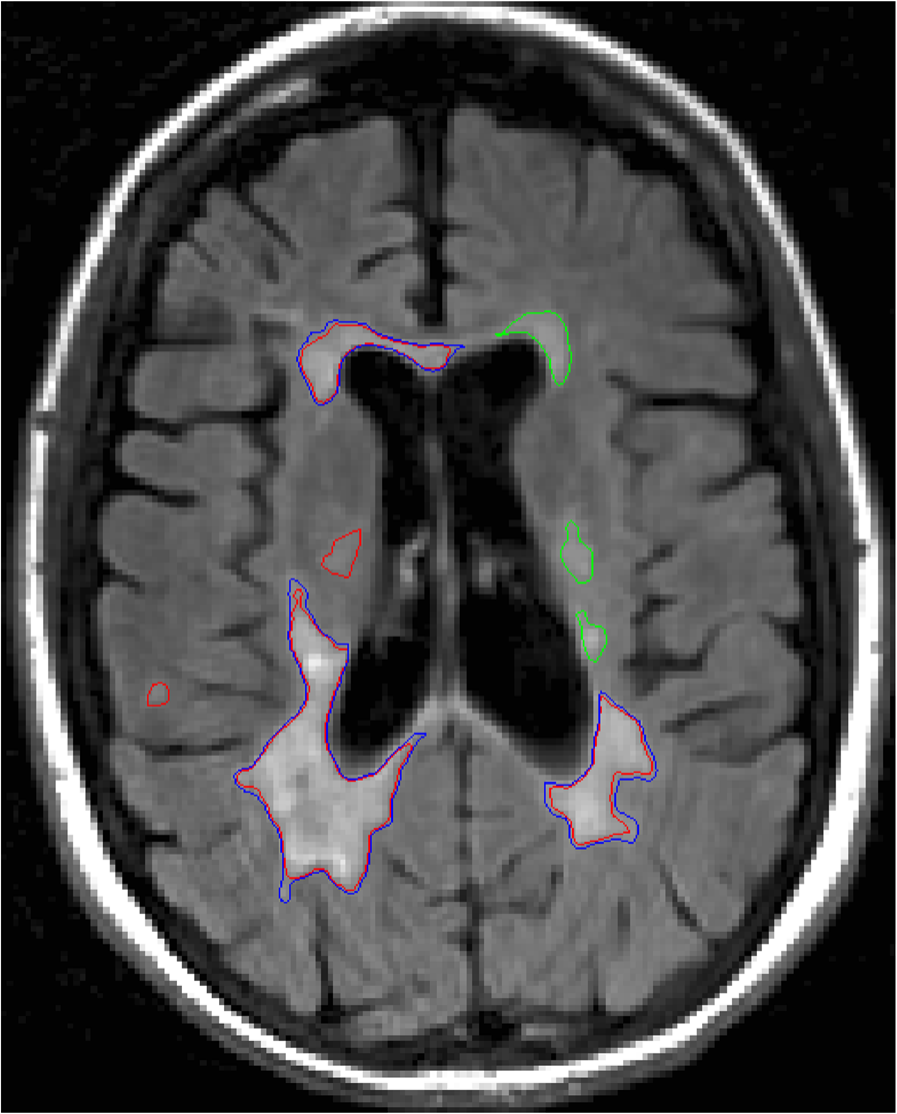
**The ROI sets from two raters are shown for a FLAIR MRI slice of a patient with MS.** The blue ROIs are from one rater and the red ROIs are from the other. The ROIs in green designate where the two raters drew the exact same ROIs. Clockwise, starting from the upper left most lesion, the sizes of the lesions were: 106.7, 131.8; 58.0, 58.0; 32.1, 32.1; 27.7, 27.7; 174.7, 224.0; 507.9, 574.6; 10.6,0; and 28.9, 0 mm^2^ for the Red and Blue raters‘ ROIs, respectively; the green ROIs were included as both Red and Blue ROIs, and 0 is used when the rater didn’t draw an ROI at that location. Although DE and OER are calculated for an entire volume, for demonstration, we find DE for this slice is 39.5 mm^2^ and OER for this slice is .142.

Mean MTA for the 17 scans was 5028 mm^2^ (SD = 1479, median = 3289 mm^2^, minimum = 848 mm^2^, maximum = 17996 mm^2^ ). 1710 CRs were formed with the average size 64 mm^2^. There were 182 CR labeled as CR1, 397 labeled as CR2, and 1131 labeled as CR12. We display two ROI sets for one FLAIR MRI slice of a patient with MS in Figure 2, to demonstrate raters’ disagreements.

**Figure 3:**
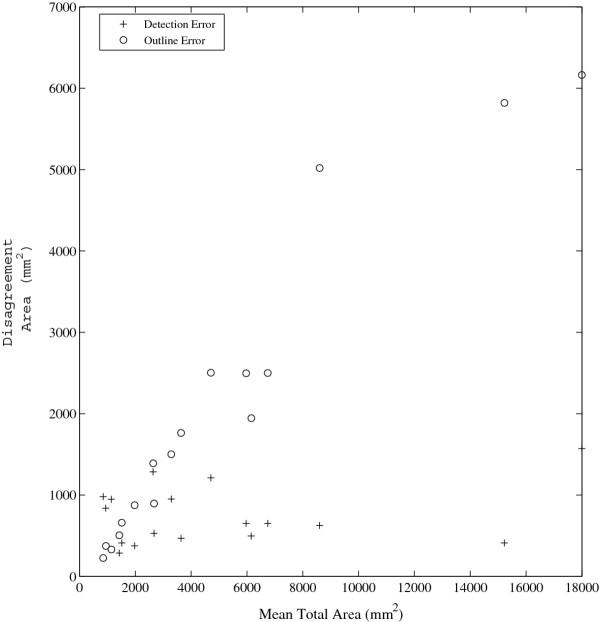
Detection and Outline Error values are plotted according to the MTA of the images.

MTA versus DE and OE values are plotted in Figure 3. While DE is relatively constant for all MTA values, OE increases with MTA. The value of OE and DE is about even for low MTA values; however, OE accounts for roughly five times more of the total error than DE for high MTA scans.

The Spearman rank correlations between MTA and SI (ρ = .75, p < .001) and MTA and OE (ρ = .98,p < .001) were significant. The rank correlations between MTA and DE (ρ = .056, p = .83), and MTA and OER (ρ = .23, p = .37) were not. Rank correlation was chosen over linear correlation for these measures because the relationship between MTA and SI is explicitly assumed to be non-linear. The mean values of DE and OER were 746.8 mm^2^, and .4077, respectively, and were used to express SI as a function of MTA:

(8) $$SI_{\mathrm{estimate}}(R1,R2)=1-\left(\frac{1}{2}\right).4077-\left(\frac{1}{2}\right)\frac{746.8}{MTA}$$

**Figure 4:**
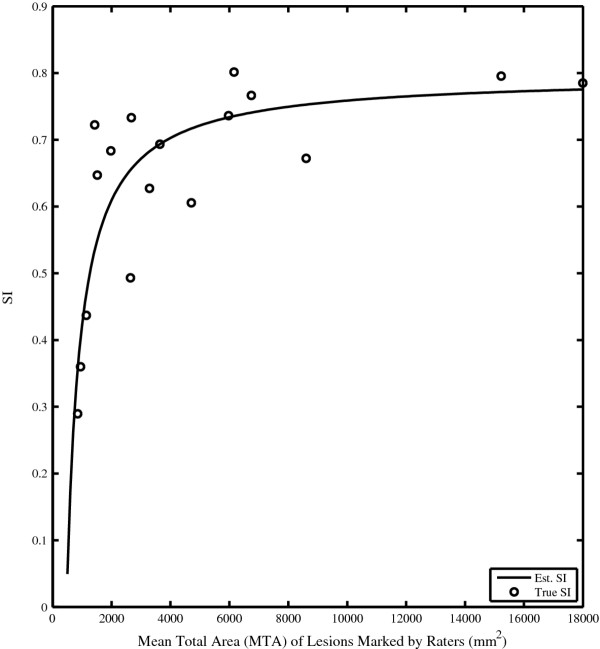
**The calculated SI values are plotted according to MTA.** The line graph relates SI_estimate_, calculated from the mean values of DE and OER, to TAA.

The calculated SI values (shown as dots) are plotted against MTA, along with the graph of SI_estimate_, in Figure 4. There was a significant (linear) correlation between SI and SI_estimate_ (r = .83, p < .001), whereas there was no correlation between the residual error and MTA (r = −.02, p = .93). An examination of the residual error did not exhibit a noticeable bias, except that the magnitude of error was clearly reduced with increased MTA. This effect indicates that there is a greater variability in rater performance for images depicting low lesion burden than high lesion burden, which is also evident from Figure 4.

Our expression for SI in terms of OER and DE provided a better fit of the measured SI values across varying lesion loads, both in an absolute and relative sense (i.e., accounting for the number of parameters used), than using the mean of the SI or a linear or quadratic fit of SI values. The sum of the square of the residual errors when fitting the measured SI values by MTA is: .383, .254, .194, and .117; for the models: mean SI value, linear fit, quadratic fit, and our DOEE method, respectively. Furthermore, the respective Akaike Information Criterion values [18] with correction for finite sample size (AICc) are: -62.19, -66.6, -68.19, and −79.78, respectively. AICc values are relative to each other and account for a varying number of parameters in competing models. The lowest AICc value indicates the model that is most likely the best model from an information theoretic perspective. Hence, the parameters OER and DE provide the best fit of SI’s dependence on lesion load, even accounting for a differing number of parameters for each model. The AICc values also allow us to calculate the likelihood one model is better than another. The likelihood that a mean, linear, or quadratic fit is better than our DOEE method is p < < .0001.

**Figure 5:**
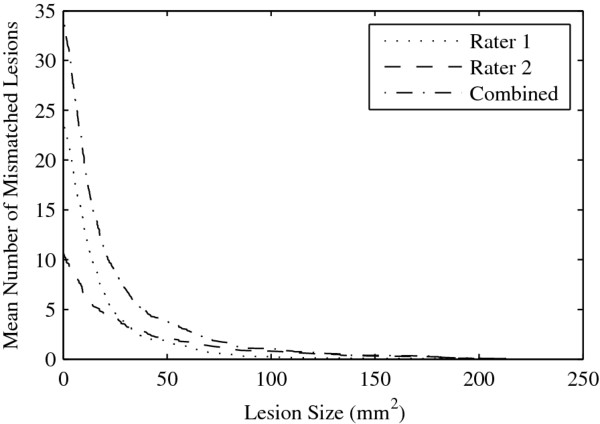
**Cumulative Detection Error plots the average number of ROI disagreements greater than a given lesion size.** Plotted are the total number of disagreements between both raters, and also individually, so that the sum of the individual disagreements equals the total disagreements.

Figure 5 is the Cumulative Detection Error Graph calculated on the set of 579 ROIs labeled as either CR1 or CR2. The average number of ROIs (per scan) missed by one rater and found by the other rater is graphed as a function of ROI threshold size, as well as the graph of the total number of missed ROIs (i.e., the sum of the two separate rater curves). The initial steep decline seen with all the curves is an indication that inter-operator agreement improves quite rapidly with increased ROI size, and then begins to level out, with a high rate of agreement (low number of errors) for larger ROIs. For example, the raters made approximately 5 detection errors (marked by only one rater) per scan for ROIs above 40 mm^2^, whereas the number of errors (mismatched ROIs) above 20 mm^2^ is much higher at approximately 20. We also observed that rater 1 drew many more lesions smaller than 40 mm^2^ than rater 2. That is, there were a greater number of CR1 regions than CR2.

We noted previously that there was not a significant correlation between MTA and OER. Furthermore, when broken down to individual ROI sizes, there was no correlation between lesion size (measured as the union of the two raters) and the intersection/union fraction of the 1131 regions of type CR12 (r = .008, p = .7883). This is an indication that rater outline agreement (as a fraction) is similar for lesions of all sizes.

**Figure 6:**
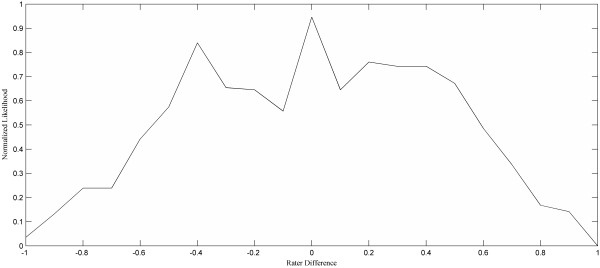
**The Outline Error Distribution graph provides an easy way of checking for outlining biases between the two raters.** Values of 0 represent identical ROIs. Negative values indicate where rater 1 drew larger ROIs; positive values give the portion of ROIs that rater 2 drew larger.

The relatively symmetric distribution, shown in the Outline Error Distribution graph in Figure 6, indicates that the two raters do not have a significant bias in how they outline a lesion. Furthermore, we are able to see that the most frequent agreement is at 0 (i.e., perfect agreement within the size of the bin), and declines in frequency as the disagreement size increases.

## Discussion

Our results confirm the observation of others that the agreement level between operators marking hyperintense MS lesions as measured by SI is dependent on the lesion burden shown in the test scans. Using AICc, which accounts for the number of parameters a model uses, the DOEE method was significantly better than the mean value, linear, or quadratic fit of the SI values (p < <.0001, for all three comparisons). Using the mean DE and OER values calculated across scans, our expression for SI in terms of MTA, $SI(R1,R2)=1-\left(\frac{1}{2}\right)OER-\left(\frac{1}{2}\right)\frac{DE}{MTA}$, has a remarkable .83 linear correlation (p < .001) with the SI values calculated for each pair of ROIs associated with a scan. Likewise, the small residual errors indicate a very good fit (Figure 4). Just as importantly, the values for DE and OER are not significantly correlated with MTA. Hence, it is easy to see that if SI measurement is used then operator agreement will appear poorest when using low lesion load scans (i.e., MTA is small, and DE/MTA is relatively large, in the equation for SI directly above), and best when using high lesion load scans (i.e. high MTA) and SI can be approximated as 1 – (1/2) OER. To summarize, while SI is commonly used to measure *rater ability*, it heavily reflects the *lesion burden* of the test set used. However, we are able to explain SI's dependence on lesion burden using just two parameters that are not dependent on lesion burden. We therefore propose these values (*mean*DE and *mean*OER) either as an addition or alternative to the reporting of mean SI values for assessing rater agreement.

The shape of the SI values plotted against MTA values (Figure 4) follows an initial steep rise followed by a leveling of values for larger values of MTA. This general shape can be observed in graphs relating SI and lesion burden from other centers [13]. The rank correlation between SI and MTA was highly significant (ρ = .75, p < .001). As values for SI are highly correlated with Kappa and JI, these later indices would also be highly dependent on the lesion burden of patients used in the test set.

Our approach divides operator differences into two types: DE and OE. These two types of errors have different characteristics. DE was predominantly constant for all scans, and had a non-significant (ρ = .056, p = .83) rank correlation with MTA. On the other hand, OE showed a strong linear relationship with MTA (Figure 3). This led to our use of OER in our equation for SI, which has a low rank correlation with MTA (ρ = .23, p = .37). OE's direct dependence on MTA is reasonable. MTA increases when there are more lesions, or the average lesion size increases. In either condition, we expect the outline error to increase. It may seem reasonable to assume a similar relationship with DE. That is, that more lesions imply operators would have a larger absolute number of differences in detecting lesions. However, this is not the case. The predominant relationship is that DE is relatively constant across scans and MTA values (Figure 3) and is well represented by a line with an intercept equal to DE and a slope equal to zero. This relationship suggests operators may have an advantage in agreeing to mark a small lesion (lower *rate* of detection error) on a scan depicting high lesion burden than a low lesion burden. That is, even though raters must mark more lesions on scans depicting high lesion volume, they will likely have the same total difference in the detection of lesions (DE) as from a scan depicting low lesion burden. We believe that DE remaining relatively constant across a range of lesion loads indicates that total size of “subtle” or ambiguous lesions remains relatively constant across scans. Outline error, on the other hand, can be well represented by a line with an intercept equal to zero, and slope equal to OER (Figure 3).

Detection error measurements, the total size (DE) and number of missed ROIs (Cumulative Detection Error graph), are especially important in the analysis of longitudinal studies. For example, a result of many ROI analyses is to establish the number of (typically small) lesions that may have newly appeared or disappeared with respect to a previous scan. In this regard, agreement measures such as SI, JI, or Kappa—or worse, operator agreement in measuring total lesion volume—are poorly suited to the task. This is especially true if the scans have a high lesion burden, since these measures are fully dominated by the raters' agreement on the outlines of large lesions. If the analysis requires the determination of small lesions, we recommend the use of the Cumulative Detection Error graph to estimate the expected number of detection errors above a given threshold size. We then recommend that a lesion threshold value be chosen for the analysis so the average number of disagreements is small.

OE is the major contributor of error by volume. While for low lesion burden the contributions of OE and DE were similar, OE was more than 5 times larger than DE for scans showing high lesion burden. As such, reducing OE (or OER) should have the greater impact on improving inter-rater measure of lesion volume. It is, therefore, not surprising that outlining of lesions using semi-automated contouring methods has been shown to reduce inter-rater variability compared to manual outlining [6]. The test for correlation between individual CR union and intersection/union was performed for 1131 CR and near zero correlation (r = .008, p = .7883) was observed. This indicates the outline agreement behaves similarly for ROIs of all sizes. The presented Outline Error Distribution graph makes use of this fact and uses ROIs of all sizes for the distribution. Even with the above findings, it is still possible that lesions with similar size will have slightly different values for the intersection/union fraction depending on the overall lesion load of the scan the lesion was from.

Breaking operator agreement into DE and OER allows an operator to be evaluated on either or both criteria according to the demands of the application. Our tests and observations provide an introduction to our developed tools for the comparison of raters creating ROIs of MS lesions. The development was driven from testing automated lesion detection methods. In this work, it quickly became apparent that the success of a method as measured by SI had little to do with the method, but instead was extensively driven by the lesion burden revealed by the images. Automated lesion detection methods are regularly reported in the literature, with their performance typically described in terms of JI, SI, or Kappa. Based on the results presented here, we see that it is difficult for the reader to compare results of different methods, since the lesion burden of the patients used to construct a test set of scans dominates how well a method performs in terms of SI. Had methods similar to ours been used, it would be relatively easy to assess the strengths and weaknesses of the different methods.

OE, DE, and SI only measure the difference between the raters, and don't distinguish between raters or a gold standard with "False Positive" and "False Negative" distinctions [19]. However, our "Cumulative Detection Error" and "Outline Error Distribution" graphs provide an informative approach—which examines whether biases exist between raters—that is consistent with our division between detection and outline differences. The initial observations made here lead to many new questions and research areas. For instance, would the incorporation of lesion contrast either with or in place of lesion size provide a better variable for the functions measuring detection and outline agreement? Additionally, our approach demonstrated usefulness for comparing rater agreement across scanning modalities, which allows us to answer questions such as: “Do raters agree better when measuring ROIs on a 3 T scanner versus a 1.5 T scanner?” Used in this way our method would be able to determine whether a hypothesized improvement is due to improved detection or outline agreement.

While we propose DE and OER as better measures for the comparison of raters' masks than using SI, JI, or Kappa, these still do not strictly measure rater performance alone. In the case of comparing 1.5 T vs. 3 T scanning modalities, this can be used as an advantage. In general, we (obviously) anticipate that raters will perform better on high quality images than on low quality images. However, our methods remove a significant confounding problem in the comparison of raters that afflicts the indices, SI, JI and Kappa. Our testing used ROI sets from two raters on 17 scans, which is more than would typically be used to evaluate a rater, and was sufficient to demonstrate the very strong correlation (r = .83, p < .001) between our estimate and true SI values. The full utility of our measures, as with SI or others, will have to be established over time, as they are used on a wider variety of applications.

## Conclusion

Like others, we have shown that SI is dependent on the lesion burden of the patients used in the test sets. However, we have provided an equation for SI's relationship to MTA, based on the calculation of the mean detection and outline errors, which did not have a significant correlation with MTA. We recommend the adoption of detection and outline error methods for the assessment of rater ROIs. Additionally, we've shown that Cumulative Detection Error and Outline Error Distribution graphs provide a center with information on where to focus efforts to improve inter-operator agreement. Based on these advantages, we argue for the use of our measurements for inter-rater agreement assessments of lesion ROIs drawn on FLAIR MRI to improve the quality of ROIs created at a center. The result is an increase in the trust of the subsequent analysis for both studies that rely on measures of total LV, and studies which are focused on individual lesion changes over time.

## Competing interest

The authors declare that they have no competing interests.

## Authors’ contributions

DSW developedthe indices, implemented the algorithm, performed data analysis,and drafted the manuscript. MGD, NB, SH, RZ, DSW designed the experiments. MGD and NB reviewed the data analysis. CDP, LR, and DR performed image analysis. GP provided MRI physics expertise for sequences and analysis. MGD, GP, RZ contributed to manuscript revisions. All authors read and approved the final manuscript.

## Pre-publication history

The pre-publication history for this paper can be accessed here:

http://www.biomedcentral.com/1471-2342/12/17/prepub
